# Role of ETS1 in the Transcriptional Network of Diffuse Large B Cell Lymphoma of the Activated B Cell-Like Type

**DOI:** 10.3390/cancers12071912

**Published:** 2020-07-15

**Authors:** Valdemar Priebe, Giulio Sartori, Sara Napoli, Elaine Yee Lin Chung, Luciano Cascione, Ivo Kwee, Alberto Jesus Arribas, Afua Adjeiwaa Mensah, Andrea Rinaldi, Maurilio Ponzoni, Emanuele Zucca, Davide Rossi, Dimitar Efremov, Georg Lenz, Margot Thome, Francesco Bertoni

**Affiliations:** 1 Institute of Oncology Research, Faculty of Biomedical Sciences, USI, 6500 Bellinzona, Switzerland; valdemarpriebe@gmail.com (V.P.); giulio.sartori@ior.usi.ch (G.S.); sara.napoli@ior.usi.ch (S.N.); echung869@gmail.com (E.Y.L.C.); luciano.cascione@ior.usi.ch (L.C.); ivo.kwee@gmail.com (I.K.); alberto.arribas@ior.usi.ch (A.J.A.); afua.mensah@ior.usi.ch (A.A.M.); andrea.rinaldi@ior.usi.ch (A.R.); emanuelezucca@yahoo.com (E.Z.); davide.rossi@ior.usi.ch (D.R.); 2 Swiss Institute of Bioinformatics (SIB), 1015 Lausanne, Switzerland; 3 Dalle Molle Institute for Artificial Intelligence (IDSIA), 6928 Manno, Switzerland; 4 San Raffaele Scientific Institute, Vita Salute University, 20132 Milan, Italy; ponzoni.maurilio@hsr.it; 5 Oncology Institute of Southern Switzerland, 6500 Bellinzona, Switzerland; 6 Molecular Hematology, International Centre for Genetic Engineering and Biotechnology, 34149 Trieste, Italy; dimitar.efremov@icgeb.org; 7 Department of Medicine A, Hematology, Oncology and Pneumology, University Hospital Münster, 48149 Münster, Germany; georg.lenz@ukmuenster.de; 8 Department of Biochemistry, University of Lausanne, 1066 Epalinges, Switzerland; margot.thomemiazza@unil.ch

**Keywords:** diffuse large B cell lymphoma, ETS1, BCL6, PRDM1

## Abstract

Diffuse large B cell lymphoma (DLBCL) is a heterogenous disease that has been distinguished into at least two major molecular entities, the germinal center-like B cell (GCB) DLBCL and activated-like B cell (ABC) DLBCL, based on transcriptome expression profiling. A recurrent ch11q24.3 gain is observed in roughly a fourth of DLBCL cases resulting in the overexpression of two ETS transcription factor family members, ETS1 and FLI1. Here, we knocked down ETS1 expression by siRNA and analyzed expression changes integrating them with ChIP-seq data to identify genes directly regulated by ETS1. ETS1 silencing affected expression of genes involved in B cell signaling activation, B cell differentiation, cell cycle, and immune processes. Integration of RNA-Seq (RNA sequencing) data and ChIP-Seq (chromatin immunoprecipitation sequencing) identified 97 genes as bona fide, positively regulated direct targets of ETS1 in ABC-DLBCL. Among these was the Fc receptor for IgM, FCMR (also known as FAIM3 or Toso), which showed higher expression in ABC- than GCB-DLBCL clinical specimens. These findings show that ETS1 is contributing to the lymphomagenesis in a subset of DLBCL and identifies FCMR as a novel target of ETS1, predominantly expressed in ABC-DLBCL.

## 1. Introduction

Diffuse large B cell lymphoma (DLBCL) constitutes the most common type of lymphoma, accounting for 30–40% of new cases each year [1]. It is a heterogeneous diagnostic category with diverse clinical, biological, and genetic presentations [1,2,3,4,5,6]. Based on transcriptional profiling, at least two main subsets are distinguished based on their cell of origin, germinal center B cell (GCB)-like DLBCL, and activated B cell (ABC)-like DLBCL [1,2,3,4,5,6], although a more subtle subdivision has been more recently proposed, integrating transcriptional profiling, whole exome sequencing, and mutational data [4,5,6]. Compared to GCB-DLBCL, ABC-DLBCL is associated with a poorer patient outcome when treated with CHOP (cyclophosphamide, doxorubicin, vincristine, and prednisone) or R-CHOP (rituximab-CHOP) therapies and, phenotypically, resembles BCR-activated B cells arrested during plasmacytic differentiation [1,2].

A ch11q24.3 region is recurrently gained in up to a fourth of DLBCL cases [7,8]. The gain is associated with the overexpression of *ETS1* and *FLI1,* two transcription factors belonging to the ETS family of proteins [7]. The role of ETS1 has been well described in different malignancies of epithelial tissues such as melanoma, breast cancer, and prostate cancer [9]. In healthy B cells, ETS1 is an important regulator of development, differentiation, antibody production, and secretion [10,11,12,13,14,15]. In particular, ETS1 mRNA is detected in pro-B, pre-B, and immature/mature B cells sorted from the bone marrow as well as in mature splenic B cells, and downregulated in antibody secreting cells [10,15,16]. In mice, the inhibition of *Bcl6*, *Pax5*, *Mitf, Ets1*, *Fli1,* and *Spib* gene expression are all involved in triggering the switch into the plasma cell phenotype by directly or indirectly repressing BLIMP1 [17]. BLIMP1 is encoded by *PRDM1* and is a master regulator of terminal B cell differentiation, often deleted or disrupted in ABC-DLBCL [2,8]. Compared to GCB-DLBCL, ABC-DLBCL presents higher expression of ETS1 [7], which is also more commonly phosphorylated at threonine (Thr) 38, as marker of ETS1 protein activation [18]. We previously suggested that the gene might contribute to blocking the differentiation toward plasma cells, for example, by inhibiting BLIMP1 [7]. Although the ch11q24.3 gain is not associated with the ABC phenotype when DLBCL are split in the two main subtypes, an enrichment for ch11q24.3 gains can be seen in the recently described DLBCL cluster 5, mainly comprising ABC-DLBCL, and cluster 2, driven by TP53 inactivation [4]. These observations suggest that ETS1 can play a relevant role in the transcriptional program of ABC-DLBCL. Thus, we studied the ETS1-regulated transcriptional network by looking at the expression changes observed after gene silencing and integrating them with ChIP-Seq data. Our data identify a role for ETS1 in the transcriptional network of ABC-DLBCL and identify the putative Fc receptor for IgM, known as FAIM3/Toso, as a major gene target of ETS1.

## 2. Material and Methods

### 2.1. Cell Lines

Cell lines were cultured under standard conditions at 37 °C in a humidified atmosphere, with 5% CO_2_. DLBCL cell lines of the ABC phenotype (U-2932, SU-DHL-2, OCI-Ly10, OCI-Ly3, TMD8, HBL1) and GCB phenotype (SU-DHL-4, SU-DHL-6, FARAGE, VAL, KARPAS422, OCI-Ly1, OCI-Ly8) were obtained and maintained as previously described [19,20], and their identity was authenticated by short tandem repeat DNA profiling (IDEXX BioResearch, Ludwigsburg, Germany). HEK293T cells used as packaging system for lentiviral production were cultured in Dulbecco’s modified Eagle medium (DMEM) GlutaMax (Invitrogen, Carlsbad, CA, USA) supplemented with 25 mM D-Glucose, 1 mM Sodium pyruvate and 10% FBS.

### 2.2. Gene Silencing

For transient ETS1 knockdown we used the Amaxa 4D Nucleofector system (Lonza, Basel, Switzerland) to introduce ON-TARGET SMARTpooled siRNA or a nontargeting siRNA as control (Dharmacon GE Healthcare, Lafayette, CO, USA). Protocols were followed according to the SG Cell Line 4D-Nucleofector X Kit L (Lonza). In brief, 2 × 10^6^ cells were prepared and resuspended in 100 µL SG solution with 500 nM siRNA or corresponding amounts of BLOCK-iT™ Alexa Fluor™ Red Fluorescent Control (Invitrogen, Carlsbad, CA, USA) as a control for nucleofection efficiency. Efficiency and cell viability were confirmed 48 h after nucleofection by flow cytometry and cells were harvested for protein lysates and total RNA extraction.

Short hairpin RNAs were obtained from the Expression Arrest The RNAi Consortium (TRC) library/Mission shRNA (short hairpin RNA) Library (Sigma-Aldrich, St. Louis, MO, USA). The shRNA plasmids used were ETS1 shRNA plasmid TRCN0000005591 (sh60D) and FCMR shRNA plasmids TRCN0000135954 (sh62C) and TRCN0000134014 (sh62D). All shRNA lentiviral plasmids were third generation pLKO.1 vectors with puromycin resistance as a selection marker. For transient transfection of HEK293T cells JetPrime (Polyplus Transfection), reagent was used. According to the manufacturer’s protocol, 2.5 × 10^6^ HEK293T cells were seeded on 100-mm plates with DMEM 24 h before transfection. For each plate, a reaction mix was prepared with the necessary plasmids: pCMV-dR8.74 packaging vector, pMD2.VSVG envelope vector and expression vector in a 1:1:5 ratio. The infection of target cells was performed as previously described [7].

Cell viability was determined using Annexin V/PI assay, following manufacturer’s protocol. Briefly, cells (2.5 × 10^5^) were stained with 5 µL Annexin-V-FITC in 195 µL binding buffer (Thermo Fisher Scientific, Waltham, MA, USA). Propidium iodide (PI) (Thermo Fisher Scientific, Waltham, MA, USA) was added to samples before analysis by flow cytometry to discriminate early and late stages of apoptosis. Acquisition of flow cytometry data was done using the BD FACSCanto system (BD Bioscience, Allschwil, Switzerland) with the FACSDiva Software (eBioscience). Analysis of flow cytometry data was done using FlowJo (Version, Treestar, City, State abbv. If USA/CA, Country).

### 2.3. RNA Extraction

RNA was isolated by Trizol (Invitrogen-Thermofisher, Waltham, MA, USA) and then DNAse was treated using RNase-free DNase Kit (Qiagen, Germantown, MD, USA). 

### 2.4. PCR Amplification and Quantitative Real-Time PCR

Total RNA extracts were reverse-transcribed using the SuperScript III First-strand Synthesis SuperMix System kit (Invitrogen) to generate cDNA (complementary DNA). Then, 800 ng of total RNA was mixed with RT Reaction Mix and RT Enzyme Mix, according to protocol. The qRT-PCR amplification was performed using the KAPA SYBR FAST qPCR Master Mix (2×) ABI Prism™ on the StepOnePlus Real-Time PCR system (Applied Biosystems, Foster City, CA, USA). All primers were designed using the web-based program Primer3Plus (http://www.bioinformatics.nl/cgi-bin/primer3plus/primer3plus.cgi) in combination with PrimerBlast for validation (https://www.ncbi.nlm.nih.gov/tools/primer-blast/). The program run on the thermal cycler was: 95 °C for 3 s, 40 cycles with 95 °C 3 s/60 °C 30 s, followed by dissociation step after denaturation and annealing. Primer efficiency was determined using linear modelling for the amplification curves with the LinRegPCR software version 2015.4 [21]. Relative quantification was calculated using the Pfaffl method [22]. Primers sequences are shown in Table S1. 

### 2.5. Western Blotting

Cells were harvested and lysed by either boiling samples in 2× Laemmli sample buffer (BioRad, Hercules, CA, USA) supplemented with β-mercaptoethanol (Merck, Kenilworth, NJ, USA) for 10 s or according to manufacturer’s protocol using M-PER buffer (Thermo Fisher Scientific) with the addition of HALT protease inhibitor cocktail (Thermo Fisher Scientific). Lysates (30–50 µg) were resolved by electrophoresis using Mini-PROTEAN TGX Precast gels (4–20% gradient or 12%, BioRad). After electrophoresis, the proteins were blotted to a nitrocellulose membrane (BioRad) by electric transfer and the membranes were blocked in TBST (20 mM Tris-HCl [pH 7.5], 150 mM NaCl, 0.1% Tween 20) with 5% nonfat dry milk (BioRad) for 1 hr at room temperature. 

The following primary antibodies were used in TBST 5% BSA (bovine serum albumin) buffer: Rabbit polyclonal α-ETS1 (C-20, Santa Cruz, CA, USA), mouse monoclonal α-ETS1 (C-4, Santa Cruz, CA, USA), rabbit polyclonal α-pT38-ETS1 (ab59179, Abcam), mouse monoclonal α-Toso/FCMR (RR-16, Santa Cruz), rabbit polyclonal α-AKT (Cell Signaling, Leiden, The Netherlands), rabbit polyclonal α-pS473-pAKT (D9E, Cell Signaling), rabbit monoclonal α-IRF4 (D43H10, Cell Signaling). The following primary antibodies were used in TBST 5% nonfat dry milk buffer: Mouse monoclonal α-GAPDH (FF26A/F9, CNIO, Madrid, Spain). The secondary antibodies used were: ECL α-mouse IgG horseradish peroxidase-linked species-specific whole antibody (GE Healthcare), ECL α-Rabbit IgG horseradish peroxidase-linked species-specific whole antibody (GE Healthcare). Membranes were treated with Westar ηC 2.0 chemiluminescent substrate (Cyanagen, Bologna, Italy) and signals were detected using digital imaging with Fusion Solo (Vilber Lourmat, Witec AG, Sursee, Switzerland).

### 2.6. Transcriptome Analysis

RNA was extracted and processed for RNA-Seq (stranded, single-ended 75-bp-long sequencing reads) using the NEBNext Ultra Directional RNA Library Prep Kit for Illumina (New England BioLabs Inc., Ipswich, MA, USA) on a NextSeq 500 (Illumina, San Diego, CA, USA), as previously described [23]. Microarray-based gene expression profiling (GEP) was done with the Illumina Whole Genome Gene Expression BeadChip, as previously described [7]. RNA-Seq was done starting from whole RNA samples with the NEBNext rRNA Depletion kit, the NEBNext Ultra Directional RNA Library Prep Kit for Illumina, and the NEBNext Multiplex Oligos for Illumina (New England BioLabs Inc.). Sequencing was performed using a NextSeq 500 with the NextSeq 500/550 High Output Kit v2 (150 cycles PE; Illumina).

### 2.7. Data Mining

Microarray data were analyzed as previously described [24]. RNA-Seq data mining was performed as previously described [25]. For functional annotation [25], microarray data were processed with regular gene set enrichment analysis while RNA-Seq data were processed with preranked GSEA (Gene Set Enrichment Analysis) on fold change-ranked values, both using the default GSEA setting. Signatures with nominal p-values < 0.05 and FDR < 0.1 were considered as biologically relevant. All expression data will be available at the National Center for Biotechnology Information (NCBI) Gene Expression Omnibus (GEO) (http://www.ncbi.nlm.nih.gov/geo) database. Publicly available expression profiles’ datasets of DLBCL clinical specimens obtained with Affymetrix Genechip U133 plus 2.0 [GSE10846 [3] and GSE31312 [26]] and Affymetrix Human Genome U133A Array (GSE4475 [27] and GSE22470 [28]) were used. The CEL raw data files were imported and preprocessed by log2 transformation with normalization using Bioconductor packages in R Studio (version 3.6). The GSE10846 dataset consisted of two separate series of specimens, which were batch corrected. For dataset with available follow-up data (GSE10846 and GSE31312), the median expression of FCMR was used as threshold to separate high- and low-expression cases for the gene of interest. The log-rank test was used to investigate the impact on overall survival of FCMR and the cumulative probability of OS (overall survival) was plotted as a curve, according to the Kaplan–Meier method using R packages’ “survival” (version 3.1-12) and “survminer” ( version 0.4.0). Multivariate analyses were performed using FCMR class (dichotomized in high and low, median expression as cutoff) and cell of origin as covariates.

## 3. Results

### 3.1. Silencing Experiments Identify ETS1-Regulated Genes in ABC-DLBCL

To identify genes and pathways regulated by ETS1 in ABC-DLBCL, we performed microarray-based gene expression profiling in three ABC-DLBCL cell lines (SU-DHL-2, OCI-Ly10, HBL1) after silencing of the transcription factor using siRNA (Figures S1 and S2). Functional annotation showed higher expression of transcripts involved in plasma cell differentiation, regulation of HIF1α targets, and genes downregulated by BCR activation in the ETS1 knock-down samples compared to controls, indicating negative regulation of these genes (Table 1 and Table S2). Conversely, the transcripts downregulated by siRNA and, thus, positively regulated by ETS1 were enriched in signatures related to BCR signaling, CD40 signaling, NFκB/TNFα pathways, immune response, and early differentiation genes (Table 1 and Table S2).

**Figure 1:**
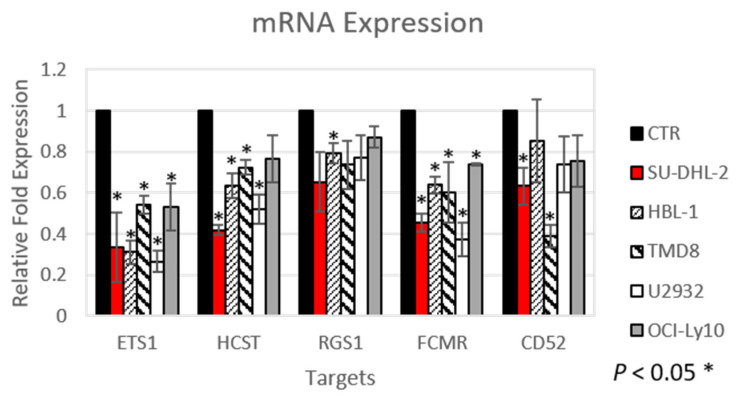
Knockdown of ETS1 reduces the expression of genes with important functions in normal B cells and DLBCL cells. A qRT-PCR was used to determine the expression of the indicated genes following knockdown of ETS1 by siRNA in five ABC-DLBCL cell lines. The expression of each gene was normalized to *GAPDH* expression. Results shown are the average of three independent experiments; *n* = 3; error bars = standard deviation. Asterisk above bars indicates significant difference in expression.

To have a better insight of the transcripts regulated by ETS1, we studied two additional ABC-DLBCL cell lines (TMD8 and U2932), this time performing RNA-Seq after ETS1 silencing by siRNA (Figure S3). Functional annotation of the results largely confirmed the data obtained in the first three cell lines, especially the ETS1 positive regulation of BCR signaling, CD40 signaling, NFκB/TNFα pathways, immune response, and HIF1α responsive genes, and the negative effect on genes involved in plasma cell differentiation (Table S3). In addition, we observed enrichment of genes involved in RNA processing following ETS1 knockdown. At gene level, 224 transcripts were differently expressed (absolute log2 fold change ≥0.2 and adj. (adjusted) *p* value < 0.05) (Figure S4, Table S3): 174 genes were downregulated and, hence, positively regulated by ETS1 and 50 genes were upregulated following ETS1 silencing and, hence, negatively regulated by ETS1. The differentially expressed genes with RNA-Seq included the ETS1 positively regulated genes *FCMR*, *RGS1*, *ARHGAP9,* and *SASH3,* which were also observed in the microarray analysis, and *TNFAIP2*, *CTTN,* and *TNIP3* among the ETS1 negatively regulated genes (Table 2 and Table S3). The *CD52* and *HCST* genes were both identified in the microarray data and were indeed expressed at lower levels after ETS1 knockdown in most cell lines, while downregulation of RGS1 was moderate in most tested cell lines and only significant in HBL1. However, when we validated by qRT-PCR, some of the genes, selected also based upon their potential relevance for DLBCL biology [29,30,31,32,33,34,35], *CD52*, *FCMR*, *RGS1,* and *HCST,* were all confirmed as downregulated after ETS1 knockdown (Figure 1). In addition, we also evaluated the expression of the known negatively regulated ETS1 target *PRDM1* [7,36], coding for BLIMP1. Following ETS1 knockdown an upregulation of PRDM1 mRNA expression was observed in HBL-1 (Figure S5), one out of two cell lines bearing the *PRDM1* gene in its wild-type configuration [8]. However, TMD8, the other cell line with wild-type *PRDM1,* did not show any increase in expression, which could be due to insufficient knockdown of ETS1 for this effect.

The ETS1 regulation of these transcripts was further validated after silencing ETS1 using shRNA in TMD8 and HBL1 cells. A downregulation in mRNA expression was confirmed for all of them (*CD52*, *FCMR*, *RGS1,* and *HCST*), as well for other genes (*PTPN7, ARHGAP9*, *SASH3,* and *GPSM3*) that had been identified only in the RNA-Seq analysis (Figure S6). However, as not all genes were significantly reduced in the TMD8 cell line, this suggests that cell-type dependency and other factors could be involved in regulating these genes.

Based on these data, ETS1 appears to control the expression of genes mainly involved in the B-cell transcriptional program but also in RNA processing. 

### 3.2. Integration with ChIP-Seq Data Identifies Putative Direct ETS1 Targets and Their Overlap with BCL6, BLIMP1, and PAX5 Targets

**Figure 2:**
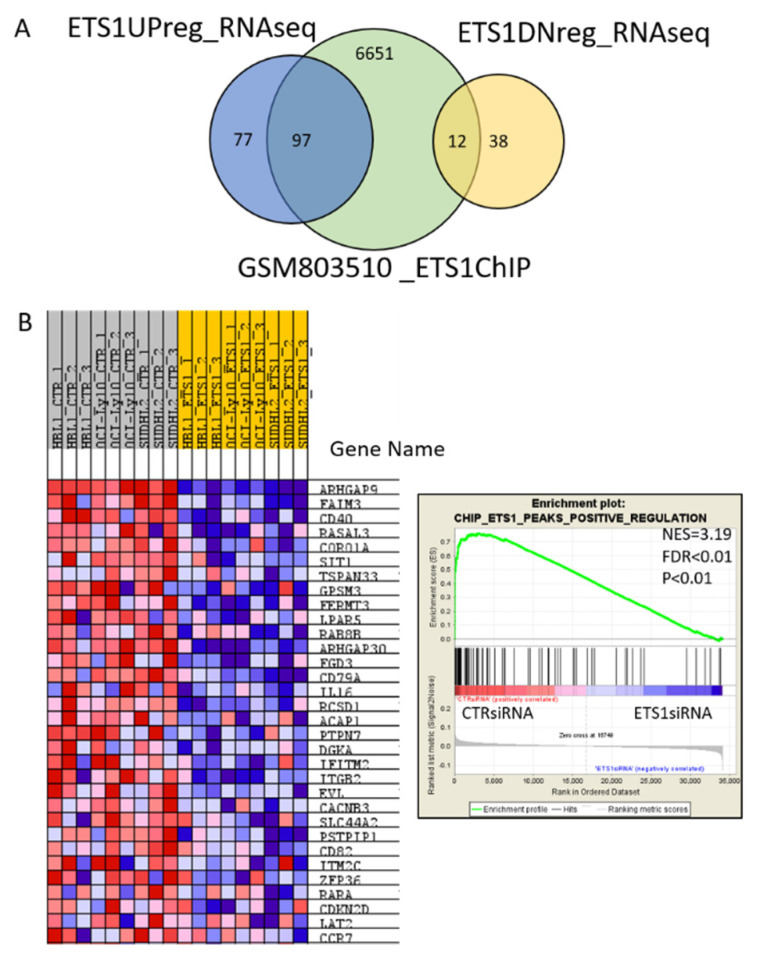
Direct ETS1 targets identified in gene expression profiling data for ABC-DLBCL cell lines with or without ETS1 knockdown. (**A**) Overlap of significant ETS1 upregulated or downregulated genes, identified from RNA-Seq data of CTR siRNA compared to ETS1 siRNA-treated TMD8 and U-2932 cell lines, with putative ETS1 targets from the publicly available Chip-Seq dataset GSM803510 obtained from B lymphoblasts. (**B**) Heatmap showing the GEP profile data (HBL1, OCI-Ly10, SU-DHL-2) for the top featured genes in the gene set for ETS1 positively regulated direct target genes identified in the RNA-Seq and ChIP-seq overlap. Expression values in the heatmap (high, moderate, low, lowest) are represented by a color range (red, pink, light blue, dark blue). In the GSEA plot: Green curve, enrichment score; bars in the middle portion of the plots show where the members of the gene set appear in the ranked list of genes; positive or negative ranking metric indicates, respectively, correlation or inverse correlation with the profile; NES, normalized enrichment score; FDR, false discovery rate.

To identify direct targets of ETS1, we took advantage of the ChIP-Seq data available at Cistrome database [37]. A list of 6760 putative ETS1 gene targets identified in human B cell lymphoblasts (Accession number GSM803510 [38]) were overlapped with the list of 224 transcripts from our RNA-Seq data. Ninety-seven genes that were identified as positively regulated by ETS1 following ETS1 knockdown, overlapped with putative ETS1 targets identified by ChIP-Seq (Figure 2A, Table S3). An overlap with putative ETS1 targets was also found for 12 of the genes we identified as negatively regulated by ETS1 (Figure 2A, Table S3). The above mentioned *ARHGAP9, FCMR*, *SASH3*, and *RGS1* were among the positively regulated. To include our microarray data in this analysis, we generated a signature of the genes that were overlapping and performed a GSEA (Figure 2B). Figure S7 shows ETS1 binding sites in four different genes (*HCST*, *FCMR*, *SASH3,* and *CD79A*) as examples.

**Figure 3:**
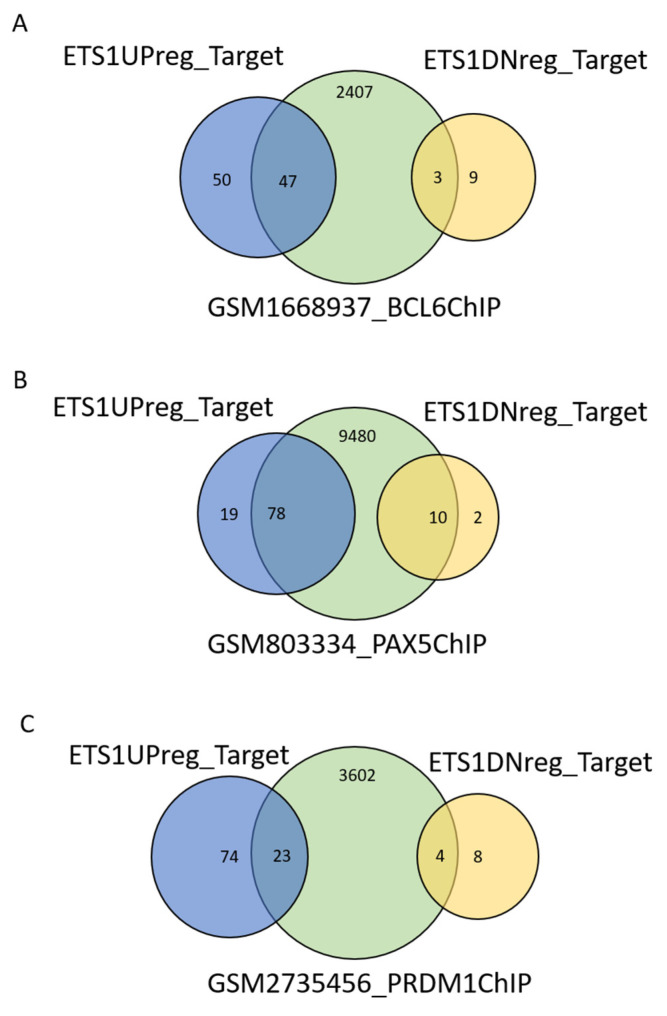
Overlap of ETS1-modulated genes with publicly available ChIP-Seq data for transcription factors important for B lymphocyte development and survival. Putative gene targets from public ChIP-Seq datasets for (**A**) BCL6, (**B**) PAX5, and (**C**) PRDM1/BLIMP1 were downloaded from the Cistrome database and overlapped with genes that we identified as modulated by ETS1 knockdown in TMD8 and U2932 ABC-DLBCL cell lines.

Exploiting three additional ChiP-Seq datasets (GSM1668937 [39], GSM2735456 [40], GSM803334 [38]), we identified direct ETS1 targets that overlap with genes regulated by BCL6, BLIMP1, and PAX5, three transcription factors that are also important for normal and neoplastic B cells (Figure 3; Table S3). Among the 97 positively regulated direct ETS1 targets, the greatest overlap was with PAX5 targets (80%), followed by BCL6 (49%): 41% of the 97 ETS1 targets were targeted by both PAX5 and BCL6. Conversely, there was only 24% overlap with BLIMP1 targets. The genes apparently co-regulated by ETS1, PAX5, and BCL6 comprised FCMR, CD40, CD79A, LMO2, PDE4A, CIITA, and IL16 among others. The overlap with PAX5 targets (83%) was the highest also among the 12 negatively regulated targets. These findings suggest that ETS1 directly participates in the transcriptional network regulated by BCL6, and PAX5 and BLIMP1.

### 3.3. The Novel ETS1 Target FCMR Is Mainly Expressed in ABC-DLBCL

The gene *FCMR*, also known as *TOSO* or *FAIM3*, was among the most downregulated genes after ETS1 silencing and presented ETS1 binding at its promoter, suggesting that *FCMR* is a putative direct target of ETS1. *FCMR,* initially described to code for an inhibitor of FAS-mediated apoptosis in T cells [41], is now recognized as coding for the Fc receptor for IgM [32,42]. 

**Figure 4:**
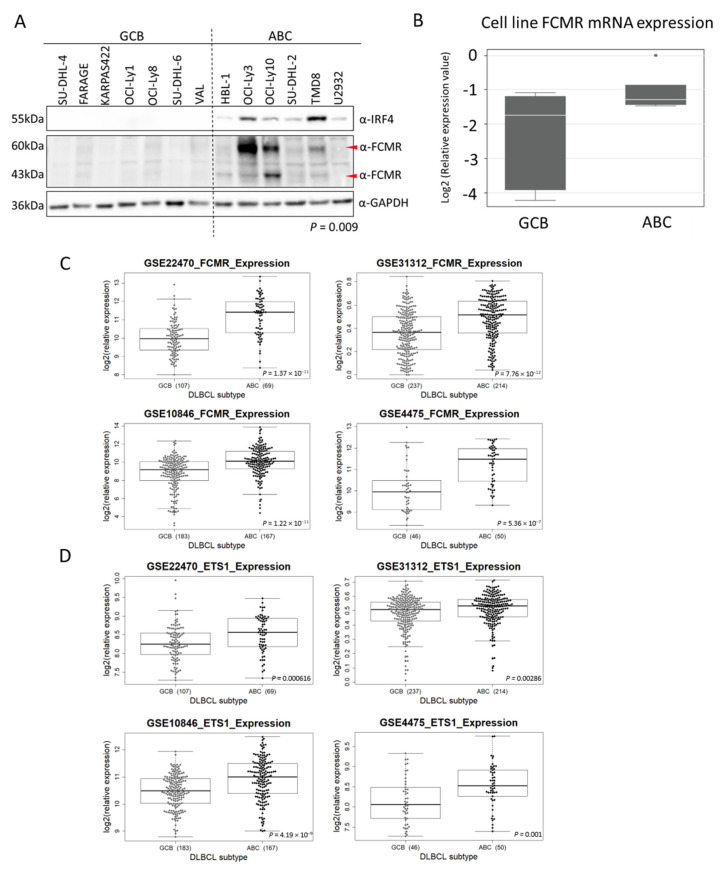
FCMR expression in clinical specimens and DLBCL cell lines. (**A**) Protein expression of FCMR in seven GCB-DLBCL and six ABC-DLBCL cell lines. FCMR (FAIM3) protein expression was significantly associated with ABC-DLBCL cell lines (Chi-square *p* = 0.009). IRF4 expression was only expected in ABC-DLBCL cell lines. The α-GAPDH was used as loading control, *n* = 3. (**B**) Quantitative real-time PCR analysis of FCMR expression in seven GCB-DLBCL and six ABC-DLBCL cell lines. Solid white lines in box-plots represent the median FCMR expression per each subset. (**C**,**D**) Differential expression of FCMR or ETS1 in four datasets comparing GCB-DLBCL to ABC-DLBCL. Expression values are Log2 transformed. Significance calculated with the Wilcoxon rank sum test. Uncropped blots See Figure S9.

ABC-DLBCL cells predominantly express IgM isotype antibodies [43], suggesting that autocrine FCMR signaling may contribute to tumorigenesis in lymphomas with ETS1-driven FCMR upregulation. Therefore, we decided to investigate FCMR expression more closely in cell lines and clinical specimens. We demonstrated that the protein was detected in cell lines derived from ABC-DLBCL (*n* = 6) while no expression was seen in GCB-DLBCL (*n* = 7) cell lines (*p* = 0.009, Figure 4A). Levels of FCMR mRNA levels were also higher in ABC-DLBCL than in GCB-DLBCL cell lines (*p* = 0.045, Figure 4B). In accordance with this observation, FCMR expression was always higher in ABC- than in GCB-DLBCL (*p* < 0.001) across different series of DLBCL clinical specimens (GSE10846 [3], GSE4475 [27], GSE22470 [28], and GSE31312 [26]) (Figure 4C). Similarly, ETS1 expression was significantly higher in ABC-DLBCL in the same datasets (Figure 4D). A high FCMR expression was associated with an inferior outcome in DLBCL patients, as assessed using two available datasets (GSE10846 [3], GSE4475 [27]). However, in agreement with the higher levels of FCMR in ABC- than GCB-DLBCL, it was not independent from the cell of origin at multivariate analyses, as also shown by the survival curves in the individual ABC or GCB subtypes (Figure S8).

**Figure 5:**
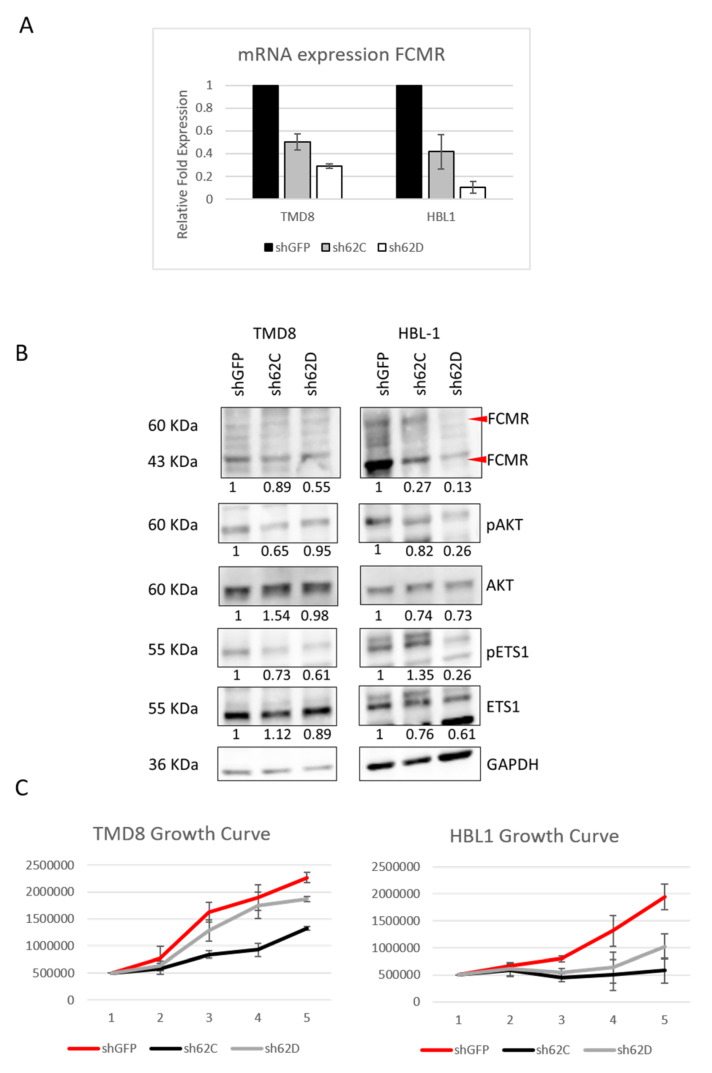
Functional analysis of FCMR knockdown in TMD8 and HBL1. (**A**) Normalized (to GAPDH) relative mRNA expression of FCMR in in TMD8 and HBL1 after knockdown using a pLKO.1 lentiviral vector expressing shRNA targeting FCMR (sh62C or sh62D) and a non-expressed gene target shGFP. (**B**) Protein expression of FCMR, ETS1, and AKT as well as phosphorylation status in TMD8 and HBL1 cell lines after FCMR knockdown. Ratios below bands are relative to control and normalized to GAPDH. (**C**) Growth of TMD8 and HBL1 over the course of five days of cell culture. Each data point represents an average of three technical replicates. Uncropped blots see Figure S10.

Since FCMR can have a modulatory effect on BCR signaling pathways [33,44,45], to see whether FCMR expression has any relevance for BCR signaling in ABC-DLBCL, we knocked down FCMR expression in two cell lines (TMD8 and HBL1) with two separate shRNAs (sh62C and sh62D) (Figure 5A). We saw a decrease in the levels of phosphorylation in pETS1 and pAKT(Ser473) in FCMR knock-down samples (Figure 5B) and there were significant differences in proliferation (but not in cell viability) in FCMR knock-down cells compared to control, if maintained in normal cell culture (Figure 5C).

## 4. Discussion

We presented a full investigation of networks regulated by the ETS1 transcription factor in ABC-DLBCL, performing transcriptome profiling after gene silencing, followed by functional annotation and integration with Chip-Seq data.

ETS1 appeared to regulate important biologic pathways: BCR activation, B cell differentiation, proliferation, and antiapoptotic pathways. These features can all be related to the main phenotype of ABC-DLBCL (constitutive B cell activation, block of terminal differentiation), indicating that ETS1 contributes to the molecular pathogenesis of this subset. The effect was largely due to a direct regulation of genes involved in these pathways, as shown by their identification as true *bona fide* ETS1 direct targets. 

Among the ETS1 positively regulated targets there were *CD79A*, ARHGAP9, *GPSM3*, *PTPN7*, *SASH3*, *HCST*, *RGS1*, *CD52,* and *FCMR*, which are part of signaling pathways known to promote B cell proliferation and survival. Together with PAX5, ETS1 interacts with the mouse *mb-1* promoter coding for CD79 [46]. Accordingly, we observed both CD79A downregulation after ETS1 knockdown as well as ETS1 enrichment at the gene promoter in publicly available ChIP-seq data. Interestingly, gain-of-function mutations affecting ITAM subunits of CD79A/B are observed in up to 20% of DLBCL patients [2,4,5,6]. Our findings, therefore, raise the possibility that ETS1 amplifies the tumor-promoting effects of these mutants by increasing their expression and, indeed, ETS1 gains are common in the ABC-DLBCL cluster 5 characterized by MYD88 and CD79A/B [4]. 

ARHGAP9 codes for a Rho GTPase, playing a role in adhesion processes of hematopoietic cells to extracellular matrix, including an inhibitory activity on MAPK signaling [47]. Due to the connection between MAP kinase cascade and ETS1 activity, this could be part of a negative feedback loop and also indicates that ETS1 might have a role in tumor cells’ migration. RGS1 is a member of the regulator of G protein-signaling family, expressed in GC B cells and lymphoma cell lines in which it desensitizes cells to chemoattractant, localizing cells to the lymph node [29]. RGS1 expression also correlates with ABC-DLBCL and with poor prognosis [30]. CD52 is a glycoprotein expressed on T and B cells, with a still-unclear function. CD52 is expressed in 75% of DLBCL cases and is downregulated in plasma cells. CD52 is also the target of the monoclonal antibody alemtuzumab that has shown low anti-tumor activity in DLBCL patients based on a series of 11 relapsed or refractory cases, not characterized for CD52 expression [31]. HCST is an adaptor protein initially described in NK cells and T cells, which, once phosphorylated, becomes a docking site for PI3K [34,35]. GPMS3 is a protein that regulates downstream intracellular signals initiated by G protein-coupled receptors and is involved in regulation of chemoattractant-mediated migration [48]. PTPN7 is a protein tyrosine phosphatase known to dephosphorylate MAPKs in hematopoietic cells, deregulated via amplifications in leukemias but deletions in lymphomas [49]. The fact that this gene is downregulated after ETS1 knockdown could suggest that PTPN7 is a target involved in a negative feedback loop for ETS1 expression. ERK-mediated ETS1 phosphorylation can lead to increased ETS1 expression via p38 MAPK [11,12,50,51]. This pathway could potentially be inhibited by PTPN7 expression induced by ETS1. Finally, SASH3, reported to be expressed in T and B cell lymphoma cell lines, is involved in signal transduction affecting immune system development and immune response [52]. 

As already mentioned, other transcription factors important for normal and neoplastic B cells are BCL6, BLIMP1, and PAX5. The integration of ETS1 data with publicly available ChiP-Seq datasets [38,39,40] indicates that there is a high overlap of the ETS1 transcriptional network with genes regulated by these other three transcriptional factors, and especially with PAX5. This is in strong agreement with the notion that ETS1 and PAX5 closely interact at the DNA level to perform their regulatory activity [46,53,54]. Differently from GCB-DLBCL, ABC-DLBCL most commonly express the IgM isotype, attributed to a genetic disruption of the switch µ region at the IgH locus, preventing Ig class switching [43,55]. The IgM phenotype also favors block of terminal differentiation unlike the IgG expression [33]. This notion led us to select FCMR for further investigation. The *FCMR* gene codes for an immunoglobulin receptor that is highly selective for IgM and can contribute to B cell activation [32,33]. However, the biologic role of FCMR in both normal and neoplastic B cells has not been fully understood [56]. Mouse models have shown that FCMR knock-down B cells have increased germinal center formation and reduced class switch and generation of Ag-specific plasma cells [45,57]. Although in CLL FCMR is overexpressed and associated with a more aggressive disease [58], in the Eµ-TCL1 transgenic CLL mouse model, FCMR loss confers an aggressive phenotype with transformation to DLBCL [59]. We observed that FCMR-silenced ABC-DLBCL cells present lower levels of phospho-ETS1 and phospho-AKT (Ser 473) compared to control cells. These data would suggest an important role of FCMR on ABC-DLBCL signaling (in AKT/ETS1 pathway). Indeed, FCMR-silenced ABC-DLBCL cells present a lower/slower growth curve compared to control cells. Our results demonstrated an important role for ETS1 in the direct regulation of FCMR, which, in turn, creates a positive loop with the upregulation of pETS1 and pAKT. These data would suggest a positive regulation by FCMR on ABC-DLBCL signaling, data fitting with the reported reduced tonic BCR signaling in FCMR-deficient mice [60]. However, the definition of the role of FCMR in normal and malignant B cells, especially in ABC-DLBCL, will need further studies [56]. 

## 5. Conclusions

Our study showed that ETS1 regulates pathways that are fundamental for ABC-DLBCL, indicating that the gene contributes to the pathogenesis of this lymphoma subtype. Among ETS1 direct targets, we identified FCMR, which was significantly more expressed in ABC-DLBCL than GCB-DLBCL and contributes to an increased cell growth with an upregulation of pETS1 and pAKT.

## Figures and Tables

**Table 1:** Summary of GSEA results for microarray data comparing ETS1 knockdown versus control in ABC-DLBCL cell lines.

Biological Processes	NAME	NES	FDR	Source
B Cell Signaling Pathways	BASSO CD40 SIGNALING UP	2.28	<0.001	MSigDB
	NFKB UP ALL OCILY3 LY10	2.11	0.002	SignatureDB
	PID BCR 5PATHWAY	2.06	0.006	MSigDB
	B CELL ACTIVATION	1.82	0.038	MSigDB
	TIAN TNF SIGNALING VIA NFKB	1.80	0.039	MSigDB
	PI3K OVEREXPRESSION UP	1.78	0.014	SignatureDB
	ACTIVATION OF NF KAPPAB TRANSCRIPTION FACTOR	1.75	0.049	MSigDB
	WIERENGA STAT5A TARGETS GROUP2	1.63	0.087	MSigDB
	PI3K OVEREXPRESSION DOWN	−1.53	0.070	Staudt
	PID PI3KCI AKT PATHWAY	−1.76	0.076	MSigDB
	PID P38 MK2 PATHWAY	−1.86	0.045	MSigDB
Cell Cycle	REACTOME G2 M CHECKPOINTS	2.48	<0.001	MSigDB
	HALLMARK E2F TARGETS	2.23	0.001	MSigDB
	REACTOME S PHASE	2.01	0.010	MSigDB
B Cell Differentiation	MARSON FOXP3 TARGETS UP	2.33	<0.001	MSigDB
	QI PLASMACYTOMA UP	2.29	<0.001	MSigDB
	ZHAN EARLY DIFFERENTIATION GENES DN	2.26	0.001	MSigDB
	PASQUALUCCI LYMPHOMA BY GC STAGE DN	2.12	0.003	MSigDB
	POSITIVE REGULATION OF CELL DIFFERENTIATION	2.06	0.006	MSigDB
	BLIMP BCELL REPRESSED	1.82	0.012	SignatureDB
	REGULATION OF CELL DIFFERENTIATION	1.80	0.038	MSigDB
	XBP1 TARGET ALL	−1.75	0.030	SignatureDB
	ZHAN LATE DIFFERENTIATION GENES UP	−1.95	0.044	MSigDB
	TARTE PLASMA CELL vs. B LYMPHOCYTE UP	−2.11	0.016	MSigDB
	PLASMACELL GENES INDUCED BYIRF4 SPIB	−2.49	<0.001	CustomIOR
DLBCL Signatures	DLBCL CLUSTER 4 FIG5	2.01	0.004	CustomIOR
	IMMUNE DLBCL GENES	1.74	0.019	CustomIOR
	GENES DOWNREGULATED AFTER IRF4 KNOCKDOWN IN ABC DLBCL HBL1	1.60	0.037	CustomIOR
	GENES UPREGULATED AFTER IRF4 KNOCKDOWN IN ABC DLBCL HBL1	−1.68	0.024	CustomIOR
	GENES UPREGULATED AFTER SPIB KNOCKDOWN IN ABC DLBCL HBL1	−1.69	0.024	CustomIOR
	IL6, IL10 STAT3 REGULATED GENES REPRESSED BY IRF4 SPIB	−1.83	0.009	CustomIOR
Hypoxia	HIF1ALPHA 2X DOWN	1.93	0.005	SignatureDB
	WINTER HYPOXIA DN	1.77	0.044	MSigDB
Immune Processes	IMMUNE RESPONSE	2.15	0.002	MSigDB
	IMMUNE SYSTEM PROCESS	2.11	0.004	MSigDB
	HUMORAL IMMUNE RESPONSE	1.79	0.041	MSigDB
	PELLICCIOTTA HDAC IN ANTIGEN PRESENTATION UP	−1.91	0.038	MSigDB
Myc Network	KONG E2F3 TARGETS	2.31	<0.001	MSigDB
	YU MYC TARGETS DN	1.97	0.013	MSigDB
	ODONNELL TARGETS OF MYC AND TFRC DN	1.85	0.031	MSigDB
	YU MYC TARGETS UP	1.78	0.041	MSigDB
	E2F3 OVEREXPRESSION 2X UP	1.68	0.027	SignatureDB
	CEBALLOS TARGETS OF TP53 AND MYC UP	−1.81	0.062	MSigDB

The GSEA included four gene set collections (Hallmark, Curated gene sets, Gene Ontology) gene sets, Oncogenic signatures) from the MSigDatabase, as well as Staudt Laboratory signatures and our own collection of custom gene sets. Significant signatures with nominal *p*-value < 0.05 and FDR < 0.1, enriched in either the control, ETS1 expressing phenotype (positive NES values), or ETS1 knock-down phenotype (negative NES values), were considered relevant. Signatures were manually grouped into biological processes. The used microarray data consisted of three ABC-DLBCL cell lines with three biological replicates, each as controls and as ETS1 knock-down samples. NES = normalized enrichment score; FDR = false discovery rate.

**Table 2:** Summary of preranked GSEA analysis of RNA-Seq data for ETS1 knockdown.

Biological Process	Gene Set	NES	FDR	Source
B Cell Signaling Pathways	MYD88 ALL DOWN	2.12	<0.001	SignatureDB
	BASSO CD40 SIGNALING UP	1.91	0.008	MSigDB
	PID RAS PATHWAY	1.90	0.009	MSigDB
	PID IL2 STAT5 PATHWAY	1.82	0.018	MSigDB
	SIG BCR SIGNALING PATHWAY	1.74	0.039	MSigDB
	REACTOME ACTIVATION OF NF KAPPAB IN B CELLS	−1.74	0.025	MSigDB
	PI3K OVEREXPRESSION UP	−2.03	0.001	SignatureDB
	EGUCHI CELL CYCLE RB1 TARGETS	−2.13	0.001	MSigDB
	REACTOME CELL CYCLE	−2.44	<0.001	MSigDB
	REACTOME MEIOSIS	−2.63	<0.001	MSigDB
B Cell Differentiation	MORI IMMATURE B LYMPHOCYTE UP	2.06	0.001	MSigDB
	BLIMP BCELL REPRESSED	2.04	<0.001	SignatureDB
	PAX5 REPRESSED	1.97	0.001	SignatureDB
	MORI MATURE B LYMPHOCYTE UP	1.92	0.008	MSigDB
	TARTE PLASMA CELL vs. B LYMPHOCYTE DN	1.66	0.064	MSigDB
	PLASMACELL GENES INDUCED BYIRF4 SPIB	−1.80	0.009	CustomIOR
	MYELOMA TACI LOW PLASMABLAST GENE	−1.85	0.005	SignatureDB
	XBP1 TARGET SECRETORY	−1.98	0.002	SignatureDB
	MORI IMMATURE B LYMPHOCYTE DN	−2.51	0.000	MSigDB
DLBCL Signatures	IMMUNE DLBCL GENES	1.79	0.010	CustomIOR
	GENES REPRESSED BYIRF4 SPIB GCB DLBCL	1.65	0.026	CustomIOR
Hypoxia	HARRIS HYPOXIA	2.03	0.002	MSigDB
	HIF1ALPHA 2X UP	1.79	0.009	SignatureDB
	HIF1ALPHA 1.5X DOWN	−3.04	<0.001	SignatureDB
	MANALO HYPOXIA DN	−3.10	<0.001	MSigDB
Immune processes	KEGG CELL ADHESION MOLECULES CAMS	2.41	<0.001	MSigDB
	IMMUNE RESPONSE	1.96	0.006	MSigDB
	IMMUNE SYSTEM PROCESS	1.95	0.007	MSigDB
	REACTOME INTEGRIN CELL SURFACE INTERACTIONS	1.84	0.015	MSigDB
RNA Processing				
	REACTOME MRNA SPLICING MINOR PATHWAY	−1.98	0.003	MSigDB
	KEGG SPLICEOSOME	−2.04	0.002	MSigDB
	REACTOME METABOLISM OF NON-CODING RNA	−2.14	0.001	MSigDB
	REACTOME MRNA SPLICING	−2.16	0.001	MSigDB
	REACTOME MRNA PROCESSING	−2.24	0.000	MSigDB
Myc Network	ODONNELL TARGETS OF MYC AND TFRC UP	2.11	0.001	MSigDB
	MYC CHIP PET EXPR DOWN	1.90	0.002	SignatureDB
	MYC RNAI OCILY3	−1.91	0.003	SignatureDB
	HALLMARK MYC TARGETS V1	−2.70	<0.001	MSigDB
	MYC OVEREXPRESSION 1.5X UP	−2.96	<0.001	SignatureDB

Summary of GSEA result for RNA-seq data comparing ETS1 knockdown versus control in ABC-DLBCL cell lines. The GSEA included four gene set collections (Hallmark, Curated gene sets, GO gene sets, Oncogenic signatures) from the MSigDatabase, as well as Staudt Laboratory signatures and our own custom gene set collection signatures. Control samples were compared to ETS1 siRNA-treated TMD8 and U2932. Significant signatures with nominal *p*-value <0.05 and FDR < 0.1, enriched in either the ETS1 expressing phenotype (positive NES values) or ETS1 knockdown (negative NES values), were considered relevant. Signatures were manually grouped into biological processes. The used RNA-seq data consisted of two ABC-DLBCL cell lines with three biological replicates each as controls and as ETS1 knockdown. NES = normalized enrichment score, FDR = false discover rate.

## Supplementary Materials

The following are available online at https://www.mdpi.com/2072-6694/12/7/1912/s1, Figure S1: ETS1 silencing in ABC-DLBCL cell lines, Figure S2: Significant differently expressed genes in ABC-DLBCL cell lines after ETS1 knockdown identified after gene expression profiling, Figure S3. ETS1 silencing in ABC-DLBCL cell lines, Figure S4: Differently expressed genes after ETS1 knock-down identified after RNA-Seq, Figure S5: qRT-PCR of PRDM1 expression after ETS1 knockdown in ABC-DLBCL cell lines, Figure S6: qRT-PCR of differently expressed genes downregulated after ETS1 knockdown in ABC-DLBCL cell lines, Figure S7. Visualization of ETS1 chromatin profile from human B cell lymphoblast, Figure S8: Clinical outcome of DLBCL cases with high or low FCMR expression; Figure S9: Uncropped blots of Figure 4, Figure S10: Uncropped blots of Figure 5, Table S1: Primers used for validation, Table S2: Significant differentially expressed genes after ETS1 knockdown for microarray data collected for cell lines HBL-1, OCI-Ly10 and SU-DHL-2, Table S3: Significant differentially expressed genes after ETS1 knockdown for RNA-seq data for cell lines TMD8 and U2932.
