# Supplementary material for: Role of ETS1 in the Transcriptional Network of Diffuse Large B Cell Lymphoma of the Activated B Cell-Like Type
Source: Cancers (Basel). 2020 Jul 15;12(7):1912. doi: 10.3390/cancers12071912 (PMC7409072; doi:10.3390/cancers12071912)
Supplement: Supplementary file 1 [file cancers-12-01912-s001.zip › cancers-859985-Supplementary Materials.pdf]

Supplementary Materials: Role of ETS1 in the Transcriptional Network of Diffuse Large B Cell Lymphoma of the Activated B Cell-Like Type

Valdemar Priebe, Giulio Sartori, Sara Napoli, Elaine Yee Lin Chung, Luciano Cascione, Ivo Kwee, Alberto Jesus Arribas, Afua Adjeiwaa Mensah, Andrea Rinaldi, Maurilio Ponzoni, Emanuele Zucca, Davide Rossi, Dimitar Efremov, Georg Lenz, Margot Thome and Francesco Bertoni

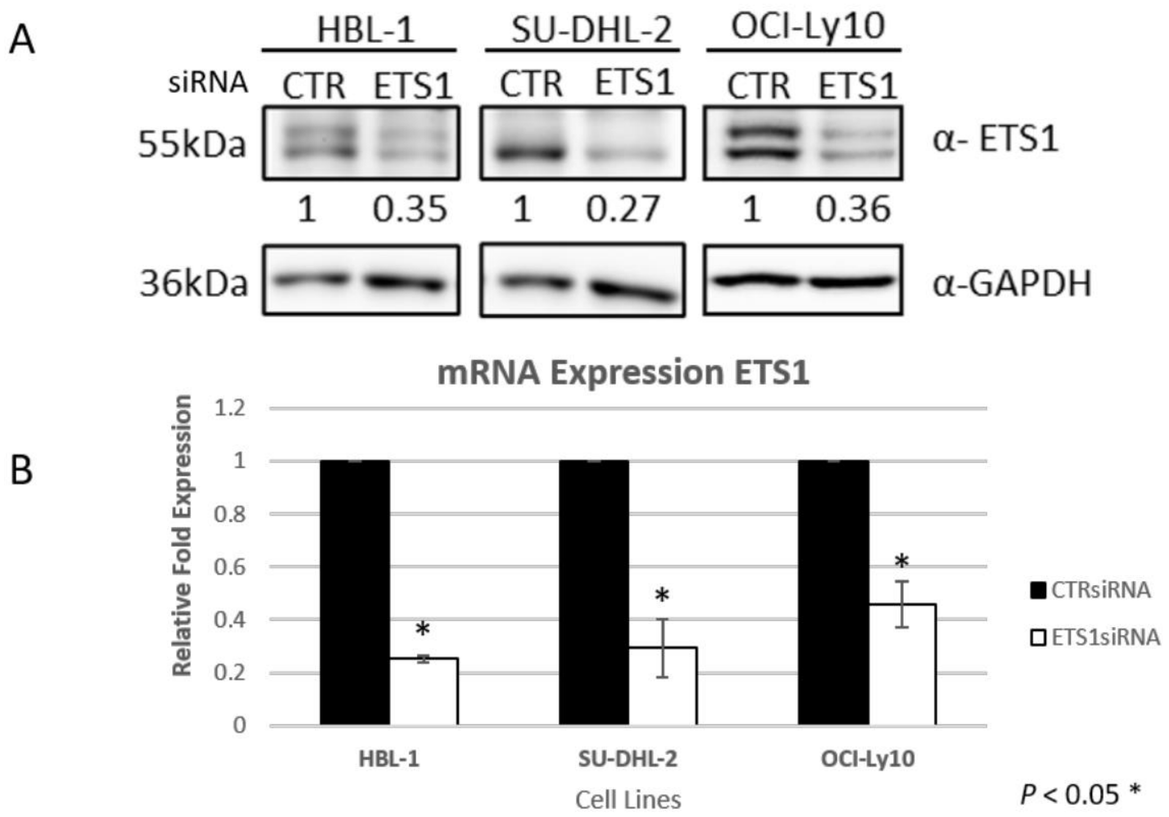

Figure S1. ETS1 silencing in ABC-DLBCL cell lines. (**A**) Immunoblot showing protein expression of ETS1 in annotated cells harvested 48 h after nucleofection with either 500 nM CTRsiRNA or SMARTpool ETS1siRNA. Mouse monoclonal α-GAPDH was used as loading control. Ratios below bands are relative to control and normalized to GAPDH. (**B**) Normalized (to GAPDH) relative mRNA expression of ETS1 from CTRsiRNA and ETS1siRNA treated cells. All images are representative for three individual experiments done for each cell line while an average relative fold change to GAPDH from three biological replicates is seen in the bar-plot diagram, $n = 3$; error bars = standard deviation. Asterisk above bars indicate significant difference in expression, $p < 0.05$.

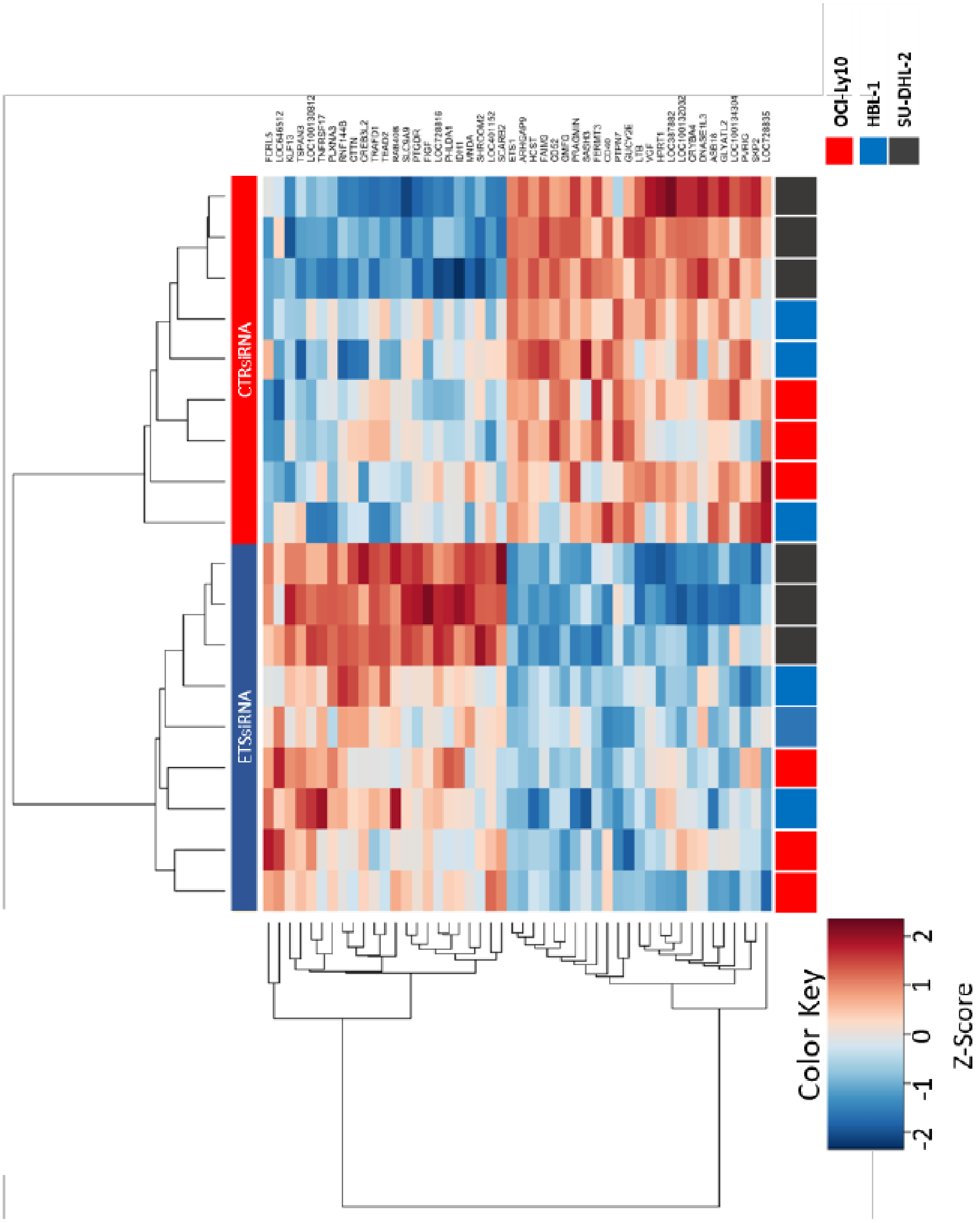

Figure S2. Significant differently expressed genes in ABC-DLBCL cell lines after ETS1 knock-down identified after gene expression profiling. Heatmap showing expression values, presented as raw Z-scores scaled from blue to red (low to high), for significant differentially expressed genes when comparing ABC-DLBCL cell lines with (ETS1siRNA, blue) or without (CTRsiRNA, red) ETS1 knockdown. Three biological triplicates of each cell line and condition were used. Cell line samples are color coded below heatmap. Genes were selected using a threshold of adj. p value < 0.05.

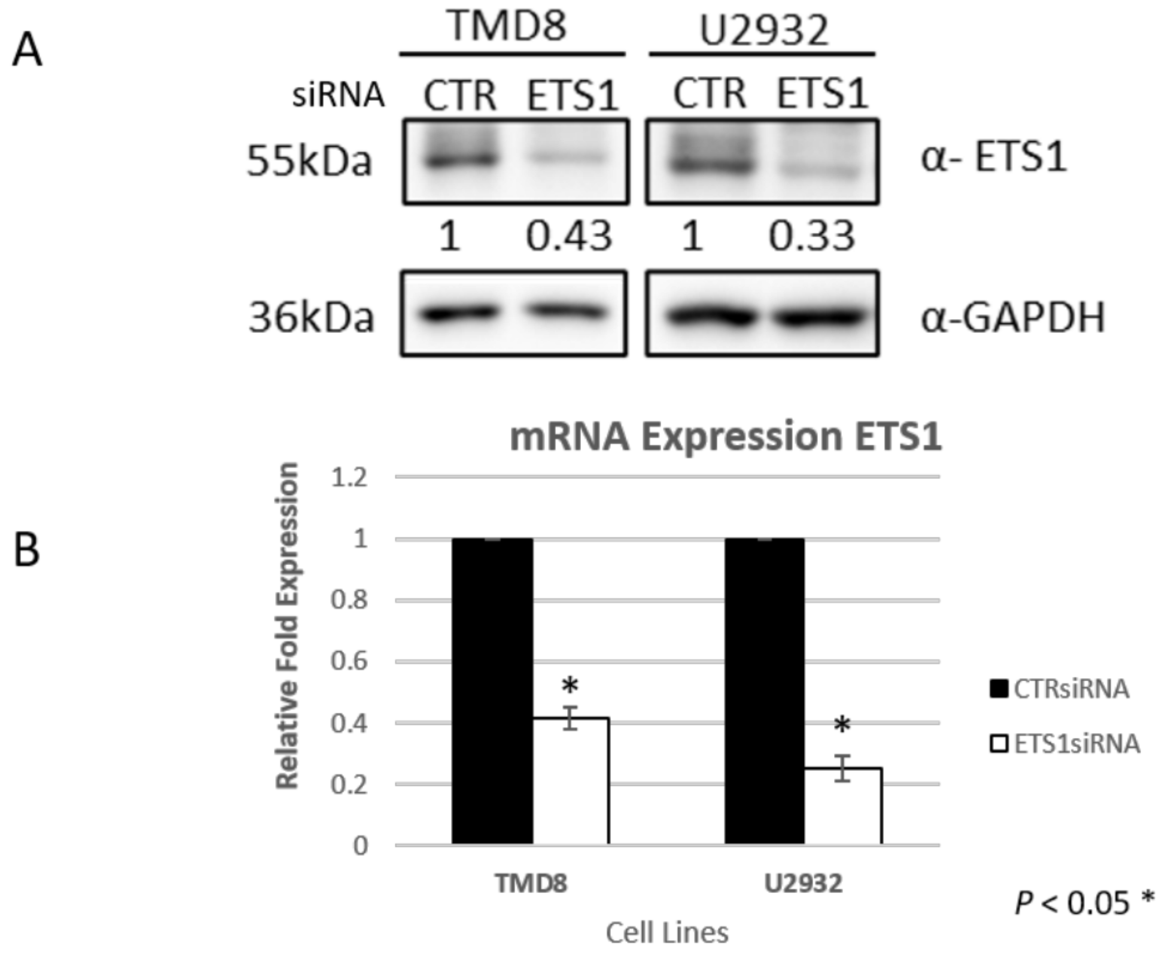

Figure S3. ETS1 silencing in ABC-DLBCL cell lines. (**A**) Immunoblot showing protein expression of ETS1 in annotated cells harvested 48 h after nucleofection with either 500nM CTRsiRNA or SMARTpool ETS1siRNA. Mouse monoclonal α-GAPDH was used as loading control. Ratios below bands are relative to control and normalized to GAPDH. (**B**) Normalized (to GAPDH) relative mRNA expression of ETS1 from CTRsiRNA and ETS1siRNA treated cells. All images are representative for three individual experiments done for each cell line while an average relative fold change to GAPDH from three biological replicates is seen in the bar-plot diagram, $n = 3$; error bars = standard deviation. Asterisk above bars indicate significant difference in expression, $p < 0.05$.

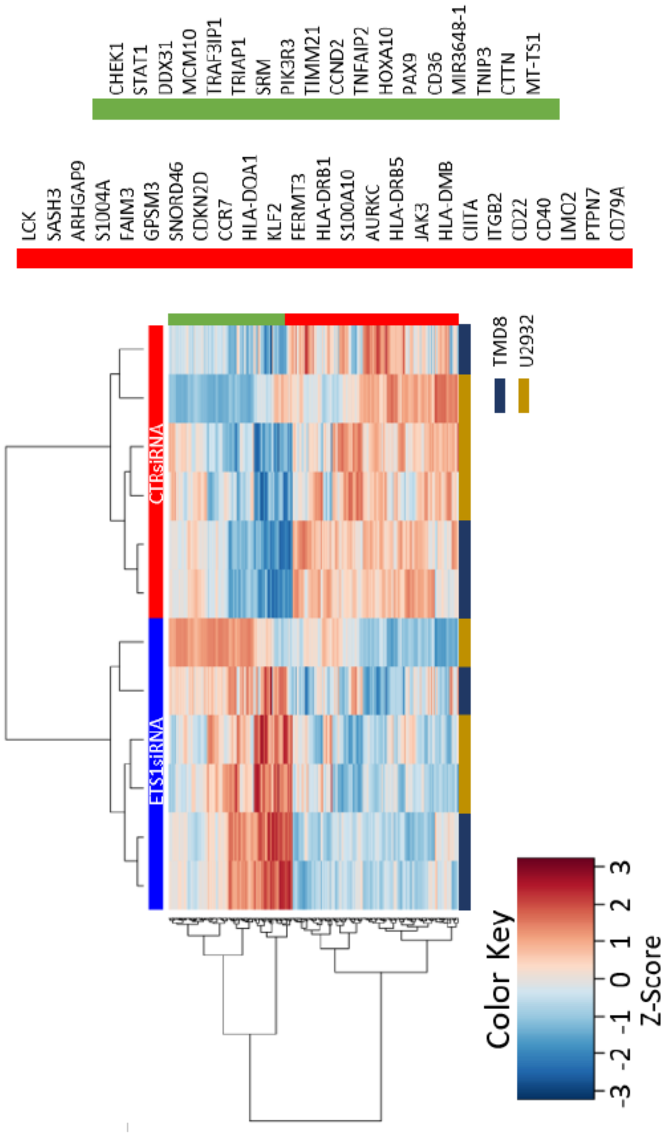

Figure S4. Differently expressed genes after ETS1 knock-down identified after RNA-Seq. Heatmap showing supervised clustering comparing cell lines with ETS1 knock-down (blue) and control (red) in U2932 (gold) and TMD8 (dark blue) cell lines. Expression values are presented as raw Z-scores scaled from blue to red (low to high), for significant differentially expressed genes when comparing ABCDLBCL cell lines with (ETS1siRNA, blue) or without (CTRsiRNA, red) ETS1 knock-down. A summarized list of the 174 significant genes downregulated after ETS1 knock-down is seen next to the red bar. Similarly, a summarized list of 50 upregulated gene are defined by the green bar.

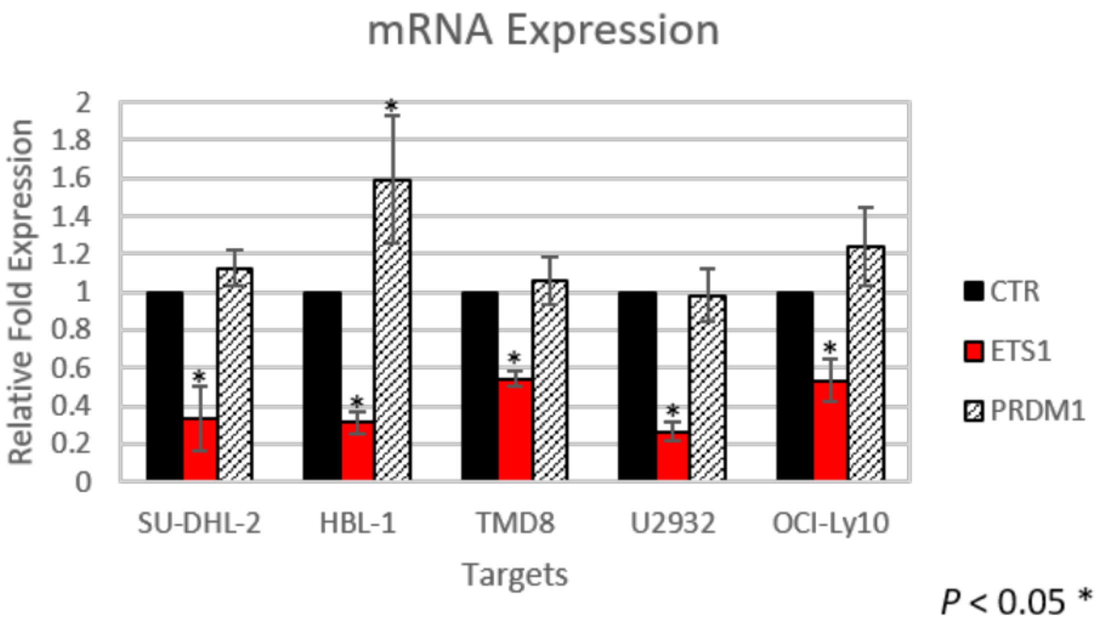

Figure S5. qRT-PCR of PRDM1 expression after ETS1 knock-down in ABC-DLBCL cell lines. Relative mRNA expression, normalized to GAPDH and to control siRNA treated cells, for genes that were significantly downregulated after ETS1 knock-down by siRNA in five ABC cell lines. Results shown are representative of three individual experiments. $n = 3$; error bars = standard deviation. Asterisk above bars indicate significant difference in expression, $p < 0.05$.

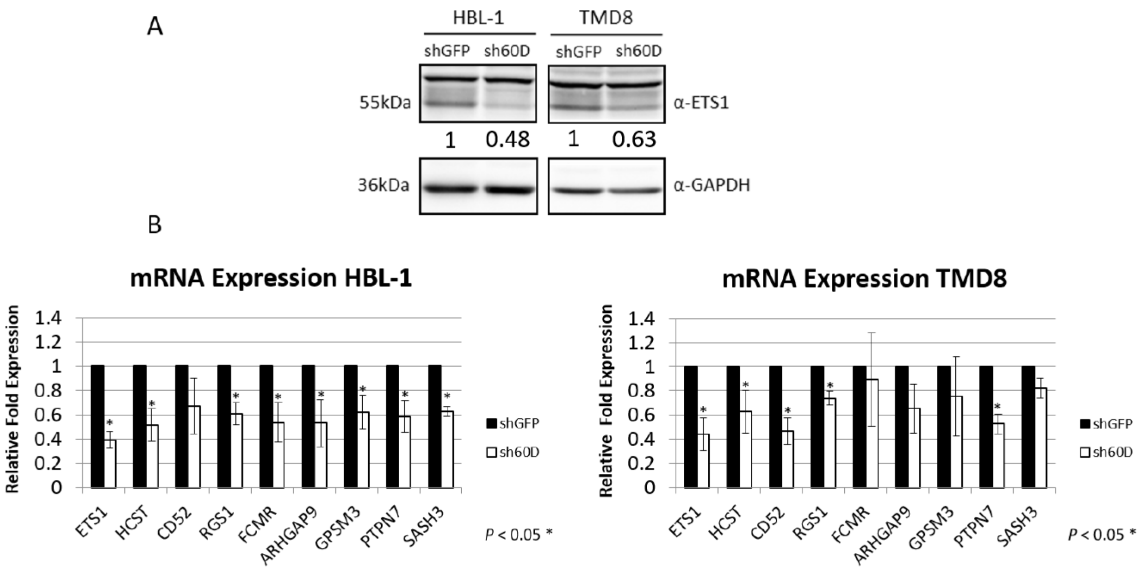

Figure S6. qRT-PCR of differently expressed genes downregulated after ETS1 knock-down in ABC-DLBCL cell lines. (**A**) ETS1 protein expression in HBL-1 and TMD8 samples transduced with a pLKO.1 ETS1 shRNA expressing vectors (sh60D). Mouse monoclonal α-GAPDH was used for loading control. Ratios below bands are relative to control and normalized to GAPDH. (**B**) Normalized (to GAPDH) relative gene expression for genes that were downregulated after ETS1 knock-down with shRNA in HBL-1 and TMD8. Results shown are representative of three individual experiments. $n = 3$; error bars = standard deviation. Asterisk above bars indicate significant difference in expression, $p < 0.05$.

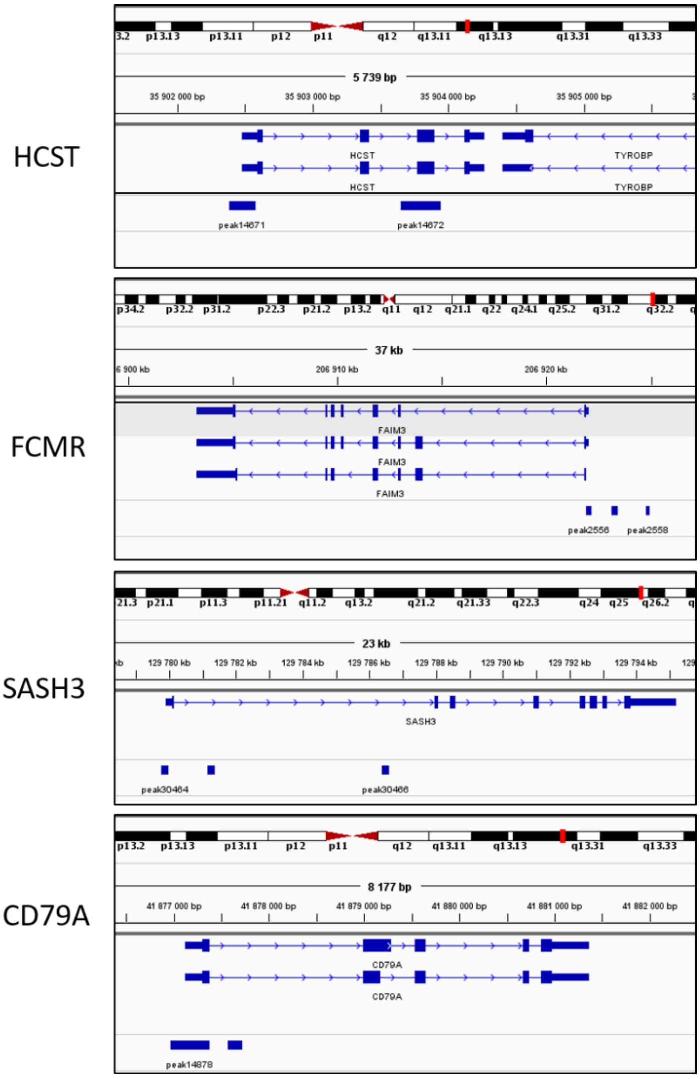

Figure S7. Visualization of ETS1 chromatin profile from human B cell lymphoblast. Visualization of BED files with called peaks for putative ETS1 targets identified in human B cell lymphoblast from the dataset GSM803510 in Integrative Genomics Viewer. Peaks were mapped to genome assembly GRCh38/*hg38* from *Genome* Reference Consortium.

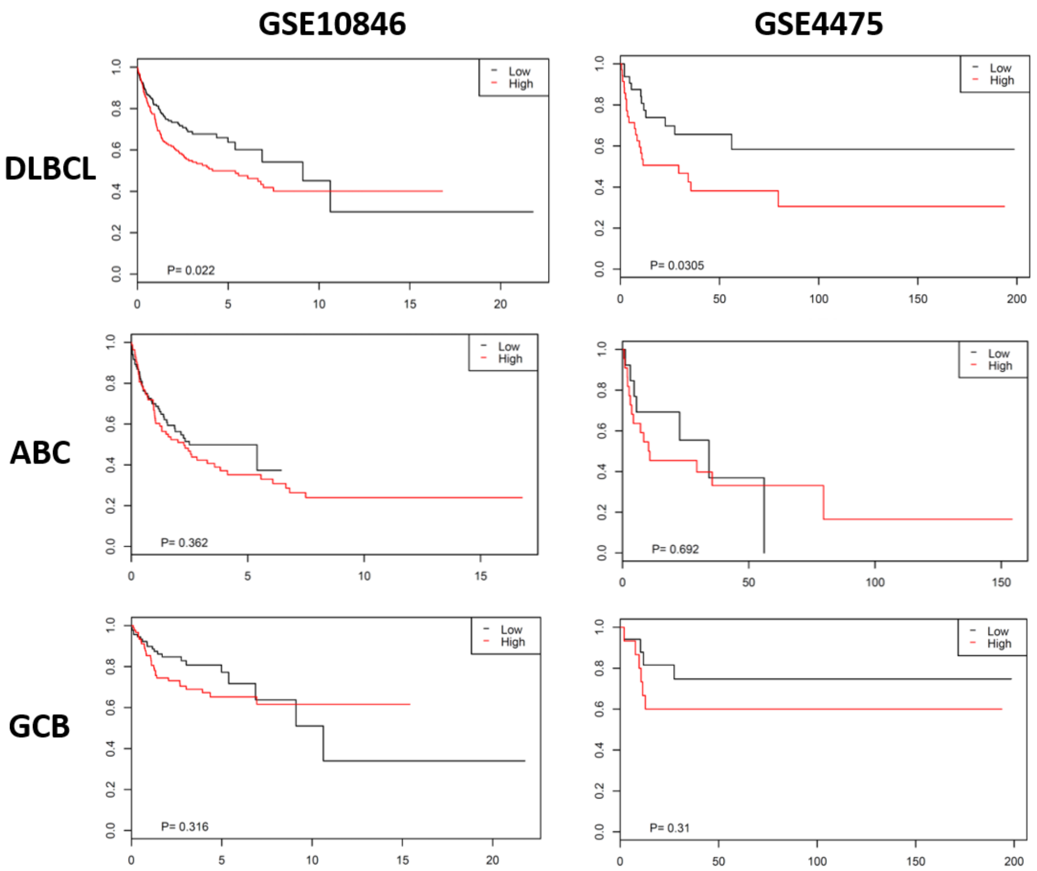

Figure S8. Clinical outcome of DLBCL cases with high or low FCMR expression. Kaplan-Meier curves were generated for two of the GSE10846 and GSE4475 DLBCL datasets with available clinical follow-up given in years or months respectively. Difference in overall survival was compared between cases with FCMR expression that was higher or lower than median. This was done for the entire datasets or for each DLBCL subtype separately (ABC-DLBCL or GCB -DLBCL). Observed significant difference in overall survival for the whole datasets is mainly caused by the difference in FCMR expression between ABC -and GCB-subtypes that has been shown earlier.

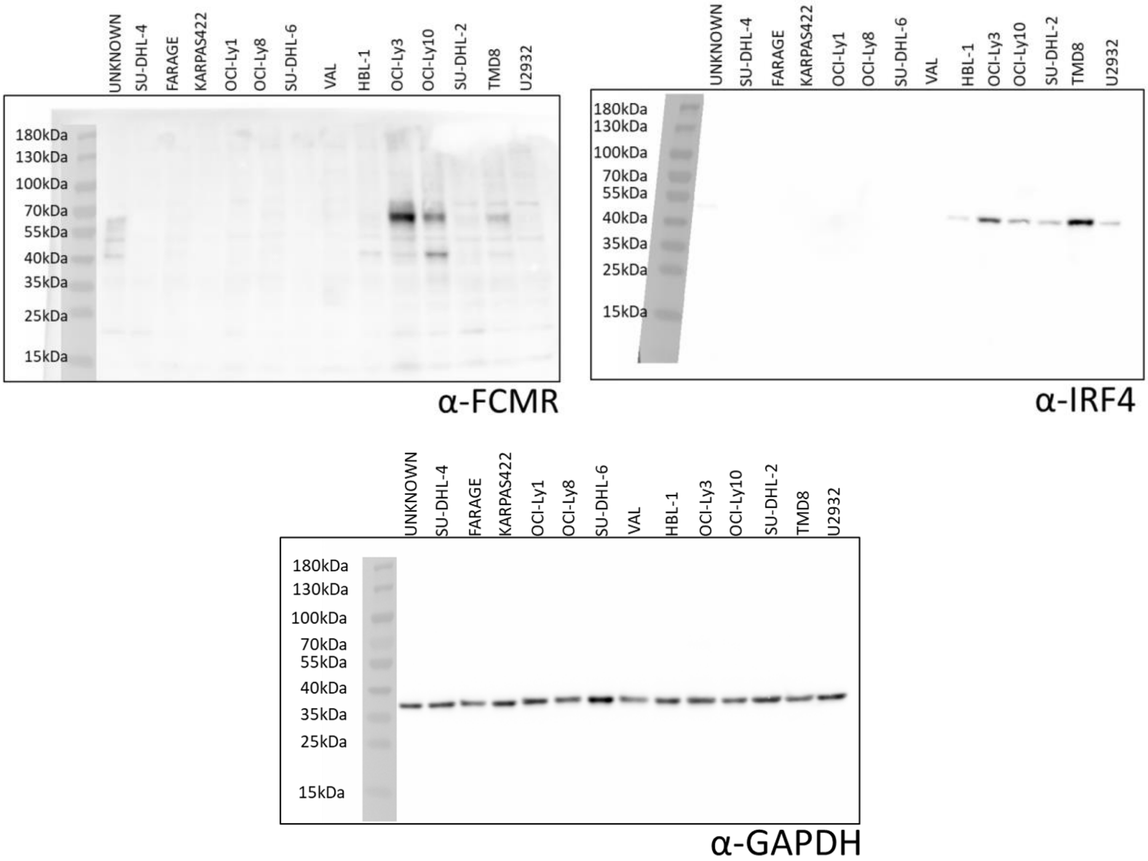

Figure S9. Uncropped blots of Figure 4. FCMR expression. Protein expression of FCMR in seven GCB-DLBCL and six ABC-DLBCL cell lines. IRF-4 expression was only expected in ABC-DLBCL cell lines. The α-GAPDH was used as loading control, n = 3. Cell line labeled UNKNOWN was excluded from final figure due to uncertainty of its identity, as some IRF4 expression was detected. Should have been DoHH2 (GCB-Subtype), that does not have IRF4 expression.

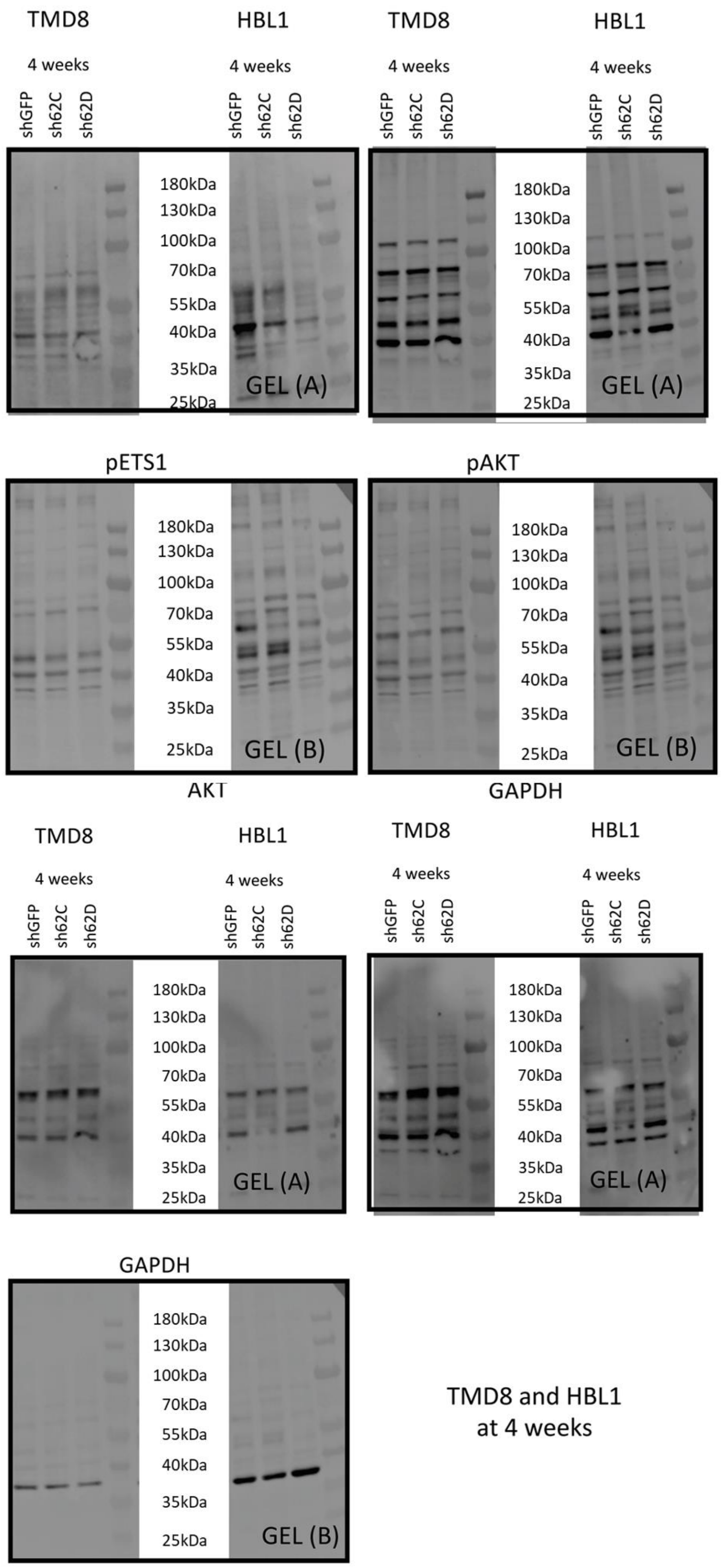

Figure S10. Uncropped blots of Figure 5. Functional analysis of FCMR knock-down in TMD8 and HBL1. Protein expression of FCMR, ETS1, and AKT as well as phosphorylation status in TMD8 and HBL1 cell lines after FCMR knock-down.

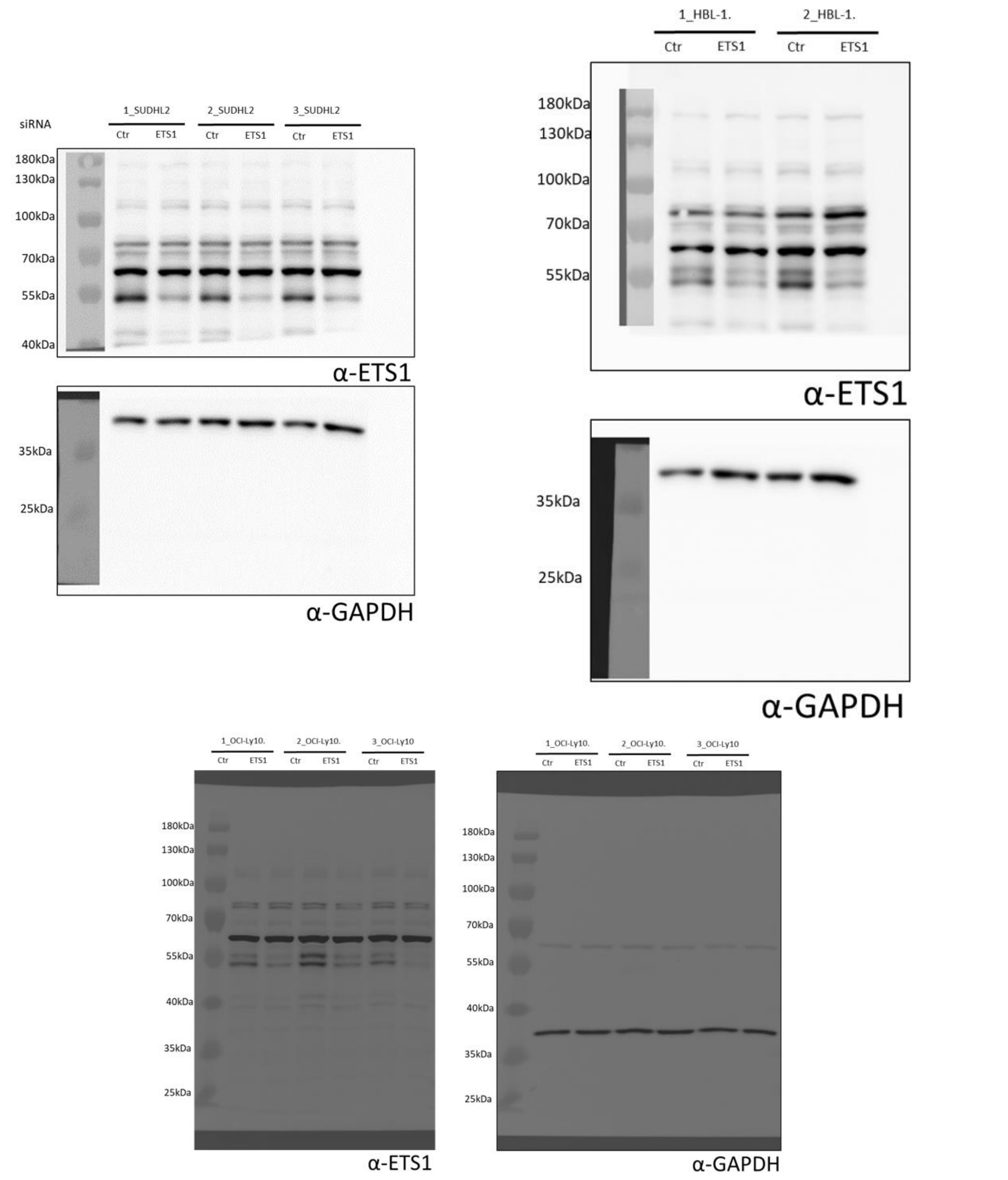

Figure S11. Uncropped blots of Figure S1 ETS1 silencing in ABC-DLBCL cell lines. Immunoblot showing protein expression of ETS1 in annotated cells harvested 48 h after nucleofection with either 500 nM CTRsiRNA or SMARTpool ETS1siRNA. Mouse monoclonal α-GAPDH was used as loading control. Some membranes were cut in half as the bottom and upper half were used for additional antibodies.

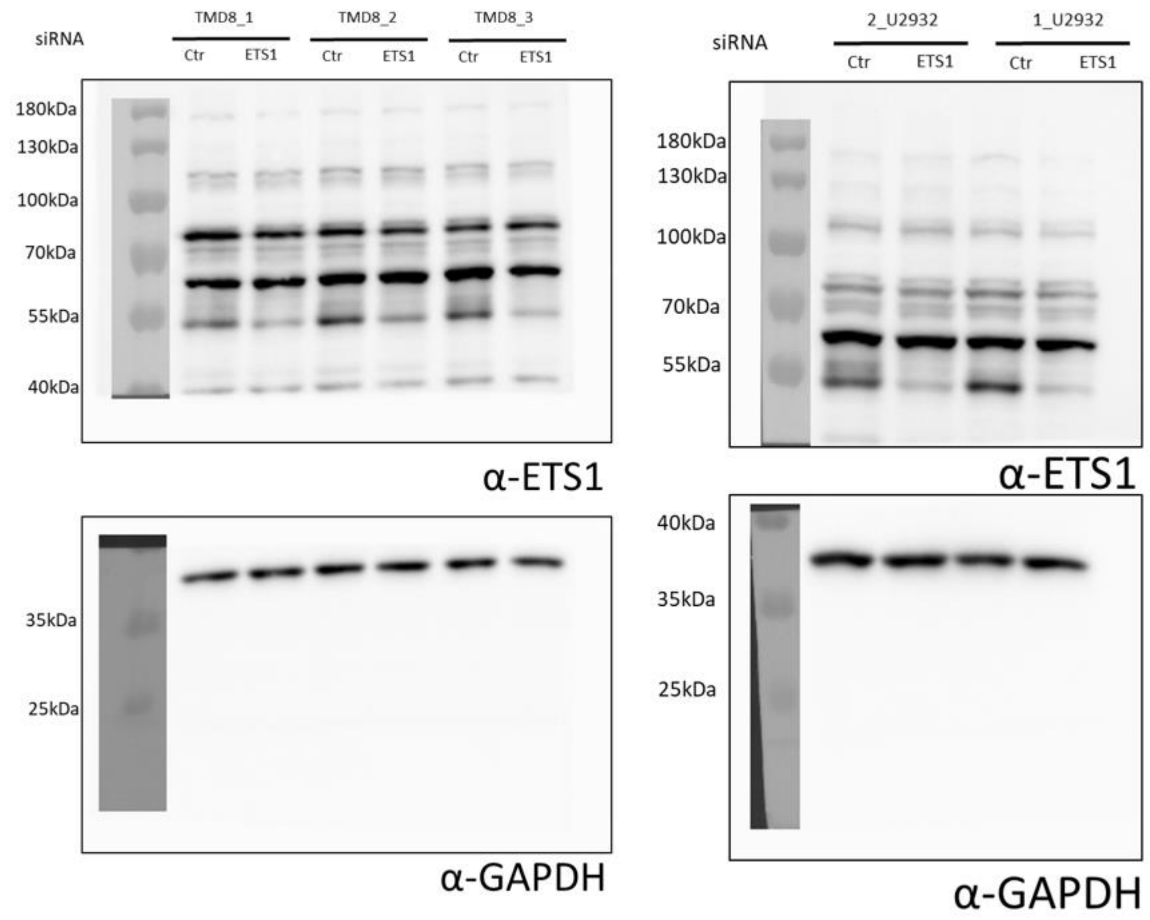

Figure S12. Uncropped blots of **Figure S3.** ETS1 silencing in ABC-DLBCL cell lines. Immunoblot showing protein expression of ETS1 in annotated cells harvested 48 h after nucleofection with either 500nM CTRsiRNA or SMARTpool ETS1siRNA. Mouse monoclonal α-GAPDH was used as loading control. Membranes were cut in half as the bottom and upper half were used for additional antibodies.

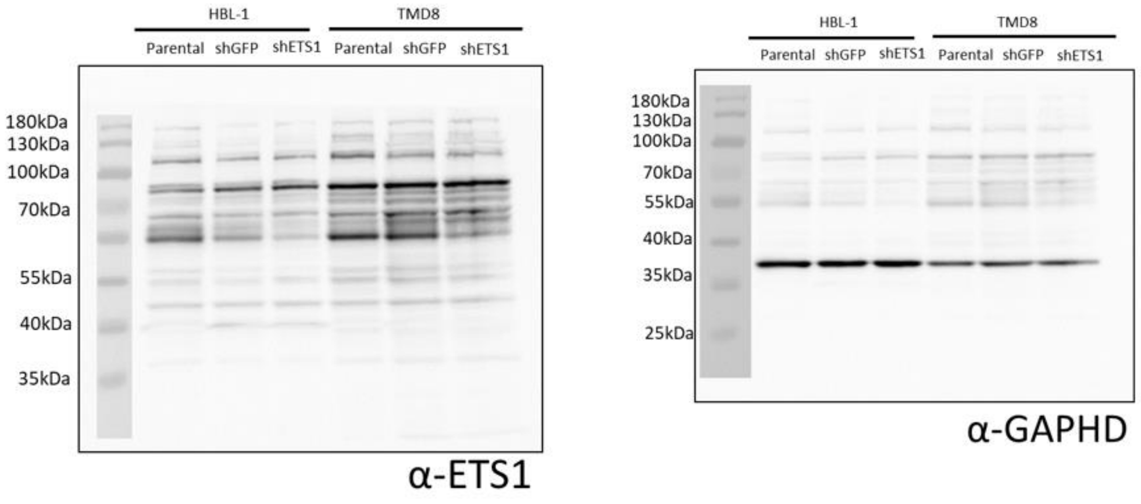

Figure S12. Uncropped blots of Figure S6. ETS1 protein expression in HBL-1 and TMD8 samples transduced with a pLKO.1 ETS1 shRNA expressing vectors (sh60D). Mouse monoclonal α-GAPDH was used for loading control.

Table S1. Primers used for validation.

**HCST**
Frw-5′- GATCCATCTGGGTCACATCCTC -′3
Rev-5′- ACCCACATCCGGAACAAGAG -′3
GPSM3
Frw-5′- TATCCTCAGTCACCAGTGCCA -′3
Rev-5′- CCTGAACTCTCAGCAGCAACT -′3
CD52
Frw-5′- CCTCCTACTCACCATCAGCC -′3
Rev-5′- TATGGCATTGGCCACGAAGA -′3
ARHGAP9
Frw-5′- AGAGACCGCCCTTACAAAGC -′3
Rev-5′- TCCCGCTCACCCGATAAATG -′3
FCMR
Frw-5′- ACTTCTGGCTTTGGCCACTT -′3
Rev-5′- CCACGGTACCACATGTTCCA -′3
RGS1
Frw-5′- GAGTTCTGGCTGGCTTGTGA -′3
Rev-5′- ATTCTCGAGTGCGGAAGTCA -′3
SASH3
Frw-5′- AGGCATCAACAGGCAGTGAG -′3
Rev-5′- CAGCGAGTCGTGGTCATAGG -′3
PTPN7
Frw-5′- AGCCAGGAGGACGGAGATTA -′3
Rev-5′- ACCATCTCCCAGAAGTCCGA -′3
GAPDH
Frw-5′- CGACCACTTTGTCAAGCTCA -′3
Rev-5′- CCCTGTTGCTGTAGCCAAAT -′3

Table S2. Significant differentially expressed genes after ETS1 knock-down for microarray data collected for cell lines HBL-1, OCI-Ly10 and SU-DHL-2. Threshold used for absolute log2 fold change of 0.2 and adjusted p value < 0.05. Additional tables show GSEA results for gene sets with nominal p-value < 0.05 and FDR < 0.1. Gene set collections used are described in Methods.

Table S3. Significant differentially expressed genes after ETS1 knock-down for RNA-seq data for cell lines TMD8 and U2932. Threshold used for absolute log2 fold change of 0.2 and adjusted p value < 0.05. Additional tables show GSEA results for gene sets with nominal p-value < 0.05 and FDR < 0.1. Gene set collections used are described in Methods.

© 2020 by the authors. Licensee MDPI, Basel, Switzerland. This article is an open access article distributed under the terms and conditions of the Creative Commons Attribution (CC BY) license (http://creativecommons.org/licenses/by/4.0/).
